# Improving Swimming Performance of Photolithography-Based Microswimmers Using Curvature Structures

**DOI:** 10.3390/mi13111965

**Published:** 2022-11-12

**Authors:** Liyuan Tan, Zihan Wang, Zhi Chen, Xiangcheng Shi, U Kei Cheang

**Affiliations:** 1 Department of Mechanical and Energy Engineering, Southern University of Science and Technology, Shenzhen 518055, China; 2 Shenzhen Key Laboratory of Biomimetic Robotics and Intelligent Systems, Southern University of Science and Technology, Shenzhen 518055, China; 3 Guangdong Provincial Key Laboratory of Human-Augmentation and Rehabilitation Robotics in Universities, Southern University of Science and Technology, Shenzhen 518055, China

**Keywords:** microswimmer, microrobot, photolithography, swimming performance

## Abstract

The emergence of robotic microswimmers and their huge potential in biomedical applications such as drug delivery, non-invasive surgery, and bio-sensing facilitates studies to improve their effectiveness. Recently, achiral microswimmers that have neither flexible nor helical structures have garnered attention because of their simple structures and fabrication process while preserving adequate swimming velocity and controllability. In this paper, the crescent shape was utilized to create photolithography-fabricated crescent-shaped achiral microswimmers. The microswimmers were actuated using rotating magnetic fields at low Reynolds numbers. Compared with the previously reported achiral microswimmers, the crescent-shaped microswimmers showed significant improvement in forward swimming speed. The effects of different curvatures, arm angles, and procession angles on the velocities of microswimmers were investigated. Moreover, the optimal swimming motion was defined by adjusting the field strength of the magnetic field. Finally, the effect of the thickness of the microswimmers on their swimming velocity was investigated.

## 1. Introduction

In the last decade, microswimmers have attracted lots of attention and have been widely investigated due to their potential bio-applications, such as drug delivery [1,2], non-invasive surgery [3,4], and cell manipulation [5]. However, these tiny microswimmers are subjected to a low Reynolds number environment where the inertial forces are negligible and the viscous forces become dominant. Investigations have shown that helical structures and flexible bodies are the two main strategies used to achieve swimming in this viscous force-dominating environment [6].

Popularized by Purcell [7], helical structures and flexible bodies have been widely used as bio-inspired design templates for many of the existing microswimmers, mimicking the swimming motion of bacteria and *spermatozoa*. The popularity of helical or flexible microswimmers is mainly because of their non-reciprocal property that breaks the time reversibility, which is known as the Scallops theory, under the low Reynolds number environment [8]. After decades of developments, many kinds of helical and flexible microswimmers have been fabricated, such as artificial bacterial flagella [9,10], the nanowire robots [11,12], and the flexible swimmers with DNA linkage [13,14].

To actuate those minimized microswimmers, externally applied power sources such as magnetic field should be often considered since on-board power such as micro/nanoscale on-board batteries is very difficult to fabricate. Recently, many methods have been developed by researchers to actuate microswimmers including light [15], heat [16], acoustic field [17], and so on [18]. Among those methods, magnetic field, especially rotating [11,14,19] and on-off fields [20,21], was widely adopted for helical and flexible microswimmers. Owing to the ability to transmit power over a long range with no health risks, a magnetic field is ideal for enabling potential clinical applications using helical or flexible microswimmers [6].

Similar to the chiral-shaped helical microswimmers, microswimmers with geometrically achiral structures were also theoretically confirmed to have the capability of swimming in a low Reynolds number environment that provides magnetic chirality [22]. These achiral microswimmers that were actuated by rotating or precessing magnetic fields have attracted much attention recently due to their extremely simple structures. The simple structures offer a substantial advantage in terms of manufacturability. For example, conventional low-cost lithography techniques can be sued to fabricate these achiral structures. Moreover, minimization of these swimmers to the nanoscale is also possible using fabrication methods like electron beam lithography and nanoimprinting. Those achiral microswimmers had either one or two planes of symmetry and presented excellent controllability and scalability [23,24]. Cheang et al. first developed achiral microswimmers with three microbeads bonded by streptavidin-biotin conjunctions. Later on, the multi-agent [25] and feedback [26] controls of those particle-based achiral microswimmers were conducted as well as theoretical investigations in the follow-up studies [27,28]. Moreover, V-shaped or L-shaped planar achiral microswimmers fabricated through photolithography were studied for their swimming performance and hydrodynamics under precession magnetic field [29,30]. These three-bead and V-shaped microswimmers are examples of magnetic chirality from the perspective of magnetic moment and actuation field, respectively. Recently, these achiral microswimmers have been explored for compatibility and the capability of drug delivery [31,32]. Even though achiral microswimmers possess swimming performances on par with swimmers with helical structures or flexible bodies, there is still a lack of research on achiral microswimmers.

In this study, crescent-shaped microswimmers were fabricated and compared with previously studied V-shaped microswimmers. The swimming performances of the fabricated crescent-shaped microswimmers are studied in different aspects. The crescent-shaped microswimmers exhibited excellent swimming performance through velocity-frequency analysis. To validate the swimming performance of crescent-shaped microswimmers, a comparison test of the swimming speed with crescent-shaped and V-shaped microswimmers of the same characteristic size was conducted. The swimming performance of the crescent-shaped microswimmers with different precession angles was studied to find the optimal condition for translational motion. In the meantime, the thickness effect on the 2D and 3D swimming performance is investigated. The studies are verified by both experiments and theoretical calculations. Finally, a zigzag swimming strategy is proposed to overcome the drifting motion of the microswimmer near the substrate. This paper aims to further explore the influence of the geometry effects on their swimming properties of achiral microswimmers.

## 2. Materials and Methods

### 2.1. Microswimmer Fabrication

**Figure 1:**
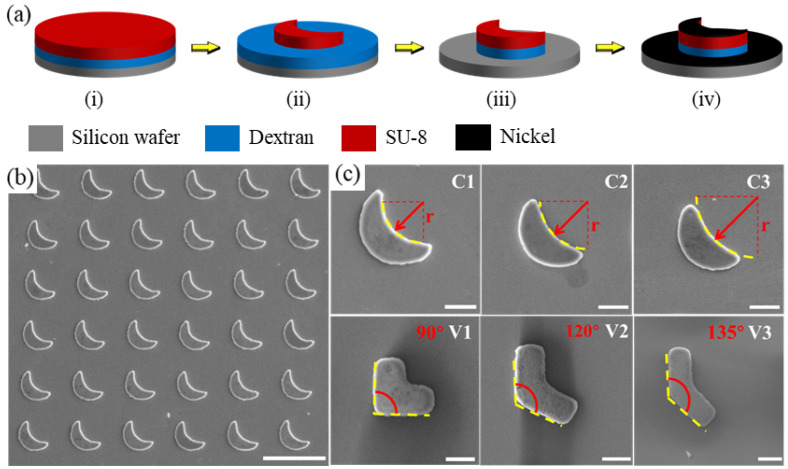
Fabrication process and geometrical design of crescent-shaped and V-shaped achiral microswimmers. (**a**) Illustration of the fabrication process of achiral microswimmers through photolithography. (**b**) Batch production of crescent-shaped microswimmers by photolithography. Scale bar: 100 μm. (**c**) C1, C2, and C3 are crescent-shaped microswimmers with different curvatures of the inner circle; V1, V2, and V3 are V-shaped microswimmers with different arm angles. Scale bars in (**c**): 20 μm.

The fabrication method, as shown in Figure 1a, of the crescent-shaped and V-shaped microswimmers involves four steps [33]: (1) spin coat water-soluble dextran sacrificial layer, (2) create crescent/V shapes via photolithography, (3) coat structures with nickel via electron beam (e-beam) evaporation to enable magnetic actuation, and (4) release microswimmers by submerging samples in water. First, a water-soluble dextran sacrificial layer was applied on a silicon wafer [34]. Then, the photoresist (SU-8 2005) was spin-coated on the dextran sacrificial layer. Crescent-shaped structures can be obtained through photolithography (soft bake, exposure, post-exposure bake, etc.). The wafer was treated by oxygen plasma to eliminate the dextran sacrificial layer that was not covered by the SU-8 structures so that the coated nickel in the next step will not be released along with the microswimmers after the fabrication. Finally, a thin nickel film (100 nm) was deposited on the SU-8 structures via e-beam evaporation giving magnetic properties to the microswimmers. For experiments, the microswimmers were released by submerging the sample in water. Since the excess dextran was removed via plasma etching, the nickel covering the area around the microswimmers will not release; this allowed the microswimmers to be suspended in a debris-free environment during the experiment. The fabrication process is shown in Figure 1a. The thickness of the fabricated microswimmers is around 4 μm except for those with different thicknesses used in Section 3.5. The microswimmers were not pre-magnetized and, therefore, the magnetic moment is in the plane of the deposited nickel film, which can be proved by having a 90° precession angle, and along the long axis (easy axis) of the patterned shape. However, unlike the microswimmer fabricated in Ref. [29] having “slim” bodies, the microswimmers fabricated in this paper are “fat” with a characteristic size on the same magnitude of the size of the entire microswimmers so that the magnetic moments are not expected to align with long axes.

### 2.2. Actuation Platform and Environment

**Figure 2:**
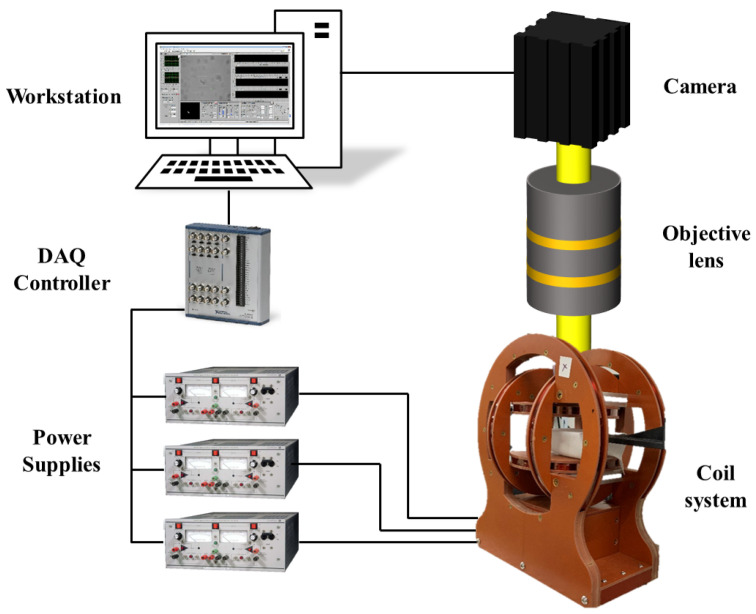
Schematic of the experimental platform generating rotating magnetic fields for microswimmer actuation.

The actuation of the microswimmers is achieved by a custom-built control system mainly consisting of a 3D Helmholtz coil system, a microscope-camera system, and a workstation. The coil system is powered by three power supplies (Kepco BOP20-5M) and controlled by a LabVIEW program using the workstation via a data acquisition device (PCI-6259, National Instrument). The velocity measurement of the microswimmers is performed in a polydimethylsiloxane (PDMS) chamber with a glass slide substrate and a coverslip cap. The experiments are conducted in water at room temperature. The experiments are performed under rotating magnetic fields that are uniformly distributed which is a well-known property of the field generated by a Helmholtz coil set. A schematic of the control system is provided in Figure 2.

The fabricated microswimmers were controlled and tested under a rotating magnetic field with different rotating frequencies and field strengths. The swimming trajectory and swimming velocity were studied and extracted by a custom-built MATLAB code tracking the position difference in centroid. All experiments to test swimming velocity were carried close to the substrate since the substrate showed negligible effect on the forward swimming velocity [30]. All the experimental data are obtained with at least five samples and the errors are standard deviations of the tested samples.

### 2.3. Theoretical Calculations

The calculations of the swimming performance of a microswimmer can be achieved by a two-step method. First, the geometry of the microswimmer is discretized by spheres characterizing the main feature of the microswimmer. Then the mobility tensors of the microswimmer, which are only related to the geometry of the microswimmer, are obtained via the multipole expansion method. Second, the swimming performance is calculated based on the combination of the exerting magnetic field, magnetic moment, and the mobility tensors of the microswimmer. Some general details of the calculation process are provided below.

First of all, the dynamics of a microswimmer are achieved by balancing the force and torque exerted on the microswimmer. The balance can be represented as [35,36],

(1) $$\left(\begin{array}{c} U\\ \Omega \end{array}\right)=\left(\begin{array}{cc} E & G\\ G^{T} & F \end{array}\right)\left(\begin{array}{c} F\\ L \end{array}\right),$$

where U and Ω are the translational and angular velocities of the microswimmer, G, F, and E are the coupling, rotational, and translational mobility tensors of the microswimmer and are only related to the geometry of the microswimmer. F and L are the forces and torques applied on the microswimmer.

Since the microswimmers we investigated in this paper are controlled by uniform fields generated by a Helmholtz coil set, the F is zero when there is no gradient field presented. This will lead a simplification of Equation (1) to [22,36,37,38]

(2) U=G·L,

(3) Ω=F·L.

Solving Equation (2) requires the knowledge of L which can be obtained by solving Equation (3). The torque L is calculated by L=m×H where m is the body-fixed magnetic moment defined in the body coordinate system (BCS) affixed to the microswimmer and H is the magnetic field defined in the laboratory coordinate system (LCS) produced by the coil set with an angular velocity of ω. The two coordinates are related by a rotation matrix R with $H^{BCS}=R\cdot H^{LCS}$. Then, Equation (3) can be solved for the torque L in the in-sync regime where the angular velocity of the microswimmer equals one of the rotating magnetic fields (Ω=ω). Plugging in the obtained L into Equation (2) gives the velocity as below

(4) $$U=R^{T}\cdot G\cdot F^{-1}\cdot \Omega^{BCS}.$$

The translational velocity can be calculated by taking the dot product of the velocity and the angular velocity which gives

(5) $$U_{Z}\cdot \omega \cdot l=\Omega^{BCS}\cdot G\cdot F^{-1}\cdot \Omega^{BCS},$$

where *l* is the characteristic dimension of the microswimmer.

The unit vector n of the magnetic moment m can be expressed by

(6) n=m/m=(sinΦcosα,sinΦsinα,cosΦ),

where *m* is the magnitude of the magnetic moment,Φ and α are the spherical polar and azimuthal angles of the magnetic moment expressed in the BCS.

Meanwhile, with a specific geometry and a fixed magnetic moment direction, the swimming performances for the microswimmers with different magnitudes of the magnetic moment and the actuation field are identical when they are scaled by a characteristic frequency $\omega_{0}=mHF_{⊥}$, where *H* is the magnitude of the magnetic field and $F_{⊥}$ is the harmonic average of the minor mobilities ($F_{1}$ and $F_{2}$) of F. Namely, the change of the magnitudes of the magnetic moment and the magnetic field will only change the frequency that achieves the specific swimming motion, but the pattern of the change of swimming motion remains the same. The detailed information on the theoretical calculations can be found in Ref. [36]. Moreover, all the curves obtained from the calculations are ended at the step-out frequency, where the microswimmers are not able to synchronize with the magnetic field, since the in-sync regime is the most interested.

## 3. Results and Discussions

### 3.1. Fabrication of Microswimmers with Curvatures

To study the effects of different geometrical parameters on the swimming performance of achiral microswimmers, both crescent-shaped and V-shaped (here we use the name after Ref. [22]) microswimmers were designed and fabricated with different curvatures and arm angles respectively. The fabricated crescent-shaped and V-shaped microswimmers shared an identical characteristic size. Figure 1b shows the batch production of crescent-shaped microswimmers with highly consistent structures obtained by photolithography. As shown in Figure 1c, three crescent-shaped microswimmers with the curvatures of 0.0385, 0.0316, and 0.0246 μm${}^{-1}$ (C1, C2, and C3) were fabricated, along with three V-shaped microswimmers with arm angle of 90°, 120°, and 135° (V1, V2, and V3).

### 3.2. Theoretical Calculations

**Figure 3:**
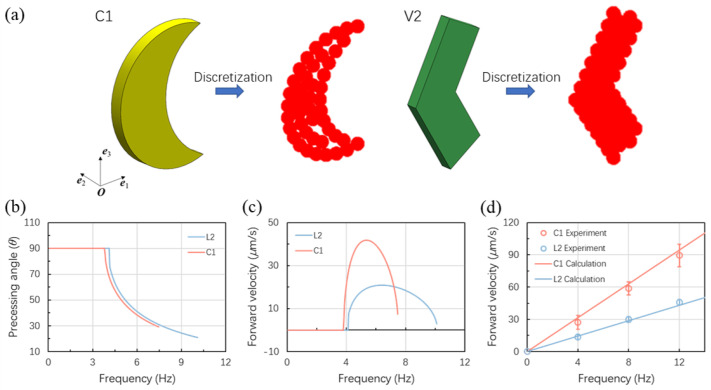
Calculation models and data. (**a**) Discretization of the C1 and V2 microswimmer into spheres. (**b**) Change of the precession angle with frequency for C1 and V2 microswimmers. (**c**) The forward velocity of the C1 and V2 microswimmers under different frequencies. (**d**) The predicted forward velocities with experimental data for C1 and V2 microswimmers with a 45° precession angle were maintained under different frequencies. Red lines and symbols are for C1 microswimmers and the blue ones are for V2 microswimmers from (**b**–**d**).

**Figure 4:**
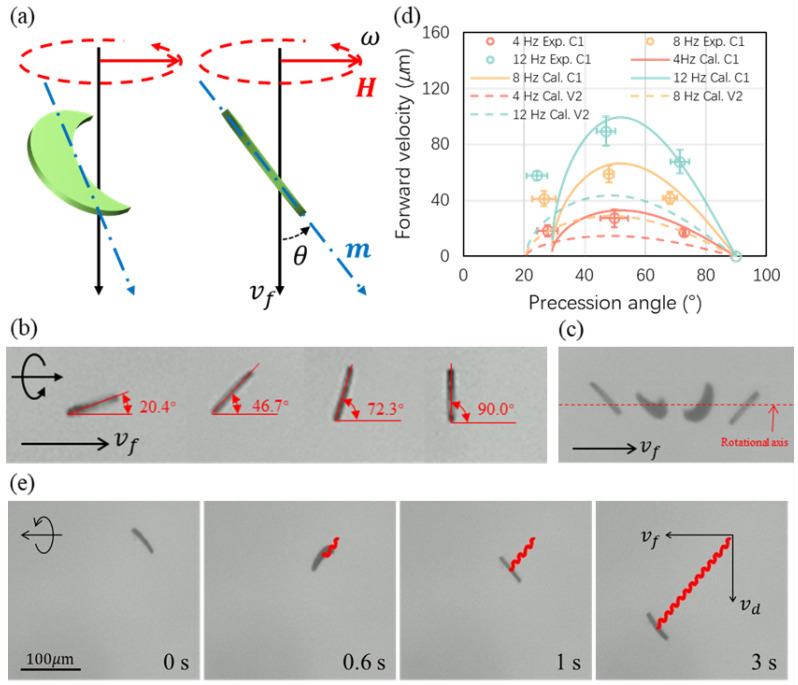
(**a**) Swimming motion of crescent-shaped and V-shaped microswimmer. The angle depicted in the figure is the precession angle; (**b**) Different swimming motions in experiments; (**c**) Swimming trajectory of a crescent-shaped microswimmer; (**d**) The effect of swimming motion or precession angle on forward swimming velocity under the different frequency of the rotational magnetic field; (**e**) Swimming with precession angle of 45° of a crescent-shaped microswimmer during a half rotation.

The theoretical calculations are achieved by first discretizing the geometry of the microswimmer into spheres. Here, the fastest microswimmers for both crescent-shaped and V-shaped microswimmers, namely, C1 and V2 are used for the calculations. The discretization of the C1 and V2 microswimmers are achieved as shown in Figure 3a. Then the mobility tensors G and F are obtained by the multipole expansion method and plugged in Equation (5) to solve for the swimming performance. The magnetic moments of the microswimmer are all in the plane of the coated nickel film and therefore the angle α is zero. The angle Φ is found for π/5 for both C1 and V2 by fitting with the experimental data presented in Section 3.4. The magnetic moment used in the calculations is 3 $\times \;10^{-10}$ emu, which is estimated by the area of the nickel coating and the saturation magnetization [39]. The precession angles and the forward velocities for C1 and V2 under a 3 mT field are shown in Figure 3b,c. From Figure 3b,c, we found that the precession angles achieving the highest velocity are around 45° for both C1 and V2. The calculation is in agreement with the experiment data as can be seen in Figure 4d.

### 3.3. The Effects of Precession Angle

Before studying the swimming performance (i.e., the velocity vs. frequency relationship) of the crescent-shaped and V-shaped microswimmers, the optimal precession angle of those microswimmers was investigated. The precession angle is defined as the angle when a planar microswimmer was vertical to the focal plane of the microscope; in other words, the angle between the plane of the microswimmer and the rotation axis as shown in Figure 4a. It was found that the rotation motion of an achiral microswimmer forms a helical trajectory as if it is part of a helical microswimmer [28]. Moreover, a helical microswimmer having a magnetic moment perpendicular to its helical axis was found to have an optimal helical angle of 45°. Therefore, a 45° of precession angle of an achiral microswimmer is a good starting point to find out the optimal precession angle of the fabricated microswimmers achieving the fastest forward velocity.

Microswimmers C1 was used to experimentally study the effect of different precession angles on the forward swimming velocity under steady swimming motion and the results were verified by calculations. The microswimmer V2 is only reported with calculations for comparison. Figure 4b presents a C1 microswimmer swimming with the precession angles of 20.4°, 46.7°, 72.3°, and 90.0°. Examples of swimming with different precession angles are provided in Supplementary Materials. The precession angle is controlled by tunning the strength of the magnetic field as the angle is an apparent behavior representing the balance between the magnetic and the viscous forces. The field strength used in this paper is on order of several mT. The steady swimming motion is characterized by the consistency of the precession angle of an entire rotation. Figure 4c shows the swimming motion of a C1 microswimmer of a half turn where the first and last images of the microswimmer with the same precession angle. Only data from steadily swimming microswimmers were used. Unsteady swimming motions can occur sometimes due to three potential factors: (i) when the swimmer encounters debris; (ii) when the swimmer reaches its step-out regime; (iii) adhesion between the boundary and the swimmers.

The results of the forward velocity with different precession angles of C1 microswimmers are presented in Figure 4d. For both C1 and V2 microswimmers, they show an optimal precession angle at around 45° to 50°. Swimming with another precession angle will have a slower speed while the calculation results are giving a step-out precession angle at around 30° for C1 and 20° for V2. The experimental data show good agreement with the calculations for higher precession angles while there is a discrepancy for low precession angles. The reason for the discrepancy can be the measurement errors for microswimmers swimming with a low precession angle with which the microswimmers were close to the step-out regime. Moreover, the inconsistency of the fabricated microswimmers may also result in the discrepancy. For example, the microswimmers may not have a magnetic moment exactly aligned in the plane of the coated nickel film, and this out-of-plane moment is having a strong effect on the overall swimming performance of the microswimmer. The error bars in Figure 4d represent standard errors that increase during measurements. Figure 4e presents a crescent-shaped microswimmer swimming with a precession angle of around 45°. When the representative achiral microswimmer was actuated by a rotating magnetic field, it had a forward velocity in the direction in alignment with its rotation axis and a drift velocity in the direction perpendicular to the forward velocity due to the rotational motion near the substrate [30].

### 3.4. Swimming Performance with Optimal Precession Angle

**Figure 5:**
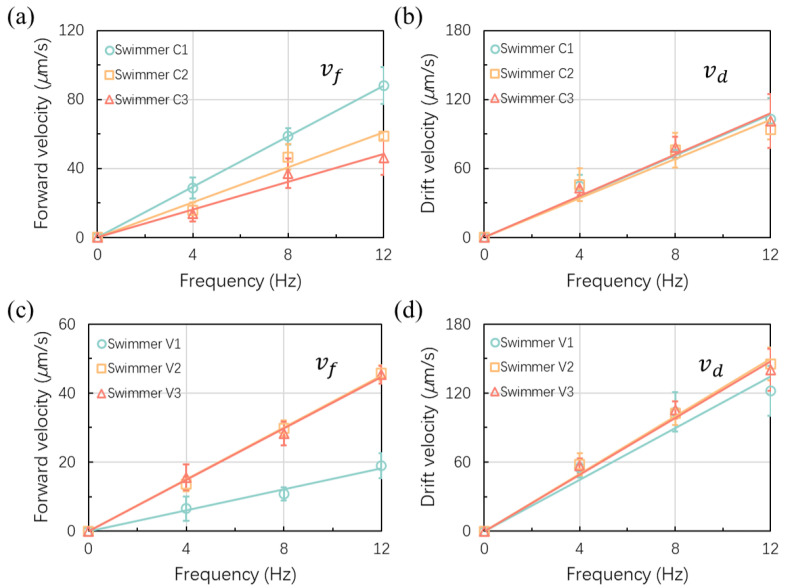
Swimming velocity of crescent-shaped and V-shaped microswimmers under the different frequencies of the rotational magnetic field. (**a**) The forward velocity of crescent-shaped microswimmers; (**b**) Drift velocity of crescent-shaped microswimmer; (in (**a**,**b**)): ☐ for microswimmer C1; △ for microswimmer C2; ◯ for microswimmer C3. (**c**) Forward velocity of V-shaped microswimmer; (**d**) Drift velocity of V-shaped microswimmers; (in (**c**,**d**)): ☐ for microswimmer V1; △ for microswimmer V2; ◯ for microswimmer V3.

As expected, the forward and drift velocities ($v_{f}$ and $v_{d}$) of both crescent-shaped and V-shaped microswimmers increased linearly with the rotational frequency when the precession angle remained unchanged, as shown in Figure 5. For crescent-shaped microswimmers, the forward swimming velocity of microswimmers with larger curvatures exhibited higher average velocities; at 12 Hz, for instance, swimmer C1’s average velocity of 89.4 μm/s is larger than C2’s and C3’s respective average velocities of 58.1 μm/s and 45.8 μm/s due to C1’s larger curvature, as shown in Figure 5a. The drift velocity of the three kinds of crescent-shaped microswimmers (C1, C2, and C3) are very similar because of their identical body length and similar precession angles, as shown in Figure 5b; thus the curvature does not affect the drift velocity. For the V-shaped microswimmers, the forward velocity with the microswimmers V2 and V3 with the respective arm angles of 120° and 135° presented similar velocities for both forward and drift velocities, as shown in Figure 5c,d. Both the forward velocity and drift velocity of microswimmer V1 with the arm angle of 90° were smaller than that of microswimmer V2 and V3. The results showed that the optimal arm angle of a V-shaped microswimmer was around 120° to 135°, which was correspondent with the results reported by Tottori et al. [29]. The average forward velocity of microswimmer V1 was 20.1 μm/s which was corresponding with Tottori’s research as well. The fastest forward velocity (45.7 μm/s) appeared when the arm angle of the V-shaped microswimmer reached 120° and the velocity was similar to the velocity between microswimmer C2 and C3 while microswimmer C1 was twice the forward velocity of the V-shaped microswimmer with an arm angle of 120°. Hence, it was obvious that the existence of curvature increased the swimming performance of an achiral planar microswimmer. Moreover, the advantage of having curvature also included the reduction of drift velocity, from 132.7 μm/s (V2) to 94.8 μm/s (C1) while the drift velocity of the V-shaped microswimmer increased with the body length when the arm angle was increased.

### 3.5. Thickness Effect and Out-of-Plane Motion

**Figure 6:**
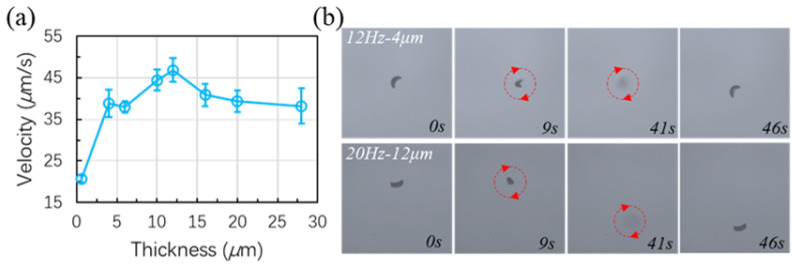
(**a**) Forward swimming velocity of the C1-type microswimmers with different thickness; (**b**) Out-of-plane (upward) motion of the C1-type microswimmers with different thickness.

The thickness effect on the translational velocity of the crescent-shaped microswimmers with a C1 design is also investigated. Various thicknesses are achieved by applying SU-8 photoresists with different viscosities or with different spin-coating speeds. All the data are obtained at precession angles close to 45°. As can be seen in Figure 6a, the forward swimming velocity is increased with the thickness until the thickness is approximating half of the critical size of the microswimmer and the velocity reached a peak. The velocity slightly decreased when the thickness was further increased. Meanwhile, the microswimmers with a thickness of 0.4 μm were not planar and had bent a little bit due to the stress of the nickel film.

Figure 6b tests the out-of-plane motion of two samples with different thicknesses under different frequencies. As can be seen from the figure, the microswimmers become blurry while going upwards. Three different microswimmers with thicknesses of 4, 12, and 20 μm are tested. However, only the microswimmers with 4 and 12 μm thicknesses can swim upward. In the meantime, the 4 μm microswimmer can start the upward swimming at 12 Hz while the 12 μm one needs 20 Hz to go upwards. The one with a 20 μm one was not able to go upwards at 20 Hz. Further increasing the frequency may help the 20 μm microswimmer but a higher frequency will result in a significantly increased viscous force which requires a much higher field strength for the force balance. The reasons for the increase of frequency in getting the microswimmers to swim upwards may be: (1) the increase of viscous force for a thicker microswimmer with a larger surface area; (2) the gravity [40] of a thicker swimmer is increased based on the thickness while the forward velocity is not increased proportionally. Therefore, a compromise between swimming velocity and other swimming properties should be considered when designing achiral microswimmers. Examples of the out-of-plane motion can be found in Supplementary Materials.

### 3.6. Discussion on the Swimming Performance

Since the viscous torque increases much faster than the magnetic torque when the frequency of the rotating field is increased, the swimming achiral microswimmer will automatically adjust its swimming motion to balance the viscous and the magnetic torque [25]. This self-adjusting manner was also predicted and observed for a helical nanomotor [41,42]. Therefore, there is a sensitive regime between the step-out regime and the regime where the field strength is sufficiently large to maintain the swimming motion, namely the precession angle, when the rotating frequency is changing. In this sensitive regime, both changing the field strength and the rotating frequency can break the torque balance and the microswimmer will automatically adjust its swimming motion to reach a new torque balance. A similar strategy has been reported for the three-bead achiral microswimmer to obtain a linearly increasing relationship between the forward velocity and the rotating frequency by keeping the ratio of the field strength and the rotating frequency. The swimming motion in this linear relationship is supposed to be the same since the thrust generated by each turn of rotation should be identical. In the experiment of this study, more magnetic torque was obtained to balance the viscous drag when field strength was sufficiently large at a specific rotating frequency. Thus the microswimmer was rolling on the boundary with a precession angle of 90°, with which the microswimmer suffered the largest viscous drag. By decreasing the field strength, the magnetic torque was decreased simultaneously so that the microswimmer must automatically adjust its swimming motion to decrease the viscous drag.

Depending on the configuration of the magnetic moment of the achiral microswimmer, two situations would occur when we keep decreasing the field strength: (i) the microswimmer encounters a step-out unsynchronized motion before the precession angle was decreased to around 0°; and (ii) the microswimmer rotates in one of the symmetrical planes when the precession angle is decreased to 0° and presents no forward swimming velocity. Therefore, we can manually decrease the step-out frequency of an achiral microswimmer or eliminate the step-out frequency by increasing the field strength. On the other hand, one can modify the field strength to adjust the precession angle of an achiral microswimmer and to obtain the optimal swimming motion. However, the resulting forward velocity is reduced at the optimal swimming motion when using a weaker field.

### 3.7. Zigzag-Trajectory with Unidirectional Swimming

**Figure 7:**
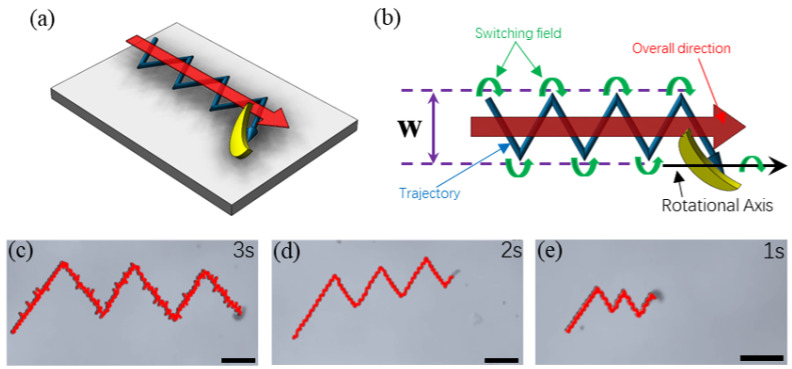
(**a**) Schematic of the zigzag trajectory strategy of a C1 microswimmer achieving overall forward swimming; (**b**) Schematic of the zigzag trajectory achieved by switching the field direction; (**c**,**d**) Zigzag trajectories achieved by a C1 microswimmer with different switching intervals of the magnetic field. Scale bars: 100 μm. (**c**) 3 s interval. (**d**) 2 s interval. (**e**) 1 s interval.

Different from the helical-structured microswimmers that could go back and forth simply by switching the rotating direction of a rotational magnetic field, achiral microswimmers will keep their forward direction when the direction of the field is reversed and the microswimmers show similar forward velocity while the direction of drift velocity was opposed. According to Cheang et al., the unchanged forward direction could be eliminated by applying a small static field [26]. When the microswimmer was rotating near the boundary, it was inevitable for the microswimmer to act with the boundary layer; thus, leading to the existence of drift velocity. Thus, by reversing the rotating direction of the rotating magnetic field, the microswimmer could swim forward along an identical direction with a switching field, as illustrated in Figure 7a,b. When applying a switching field, the rotational motion of the microswimmer is reversed with a change of the drifting velocity in direction while the direction of the forward velocity is remaining the same. Directional swimming is kept with this strategy and the width (W) of the entire trajectory can be controlled by the switching interval of the field. The zigzag swimming of a microswimmer was conducted with experiments with different reversing time intervals of 1, 2, and 3 s (in Figure 7c–e), and a difference in swimming velocity was observed. Both the forward and drift swimming velocities were found to be different as can be seen in Figure 7d (also see Supplementary Materials). This can be explained by the thickness of the SU-8 photoresist that formed the main body of the microswimmer and the single-sided and asymmetrically deposited nickel film.

## 4. Conclusions

In conclusion, the crescent-shaped achiral microswimmer was fabricated and compared with the V-shaped microswimmer. According to the results, introducing curvature could significantly improve the swimming performance, both forward and drift velocity, of an achiral microswimmer. For crescent-shaped and V-shaped with identical critical sizes, the fastest crescent-shaped microswimmer was twice the forward velocity of the V-shaped microswimmer with the optimal arm angle (120°). Meanwhile, the forward velocity with different swimming motions of the crescent-shaped microswimmer was investigated and microswimmers with around 45° to 50° precession angle were found to have the optimal swimming motion. Importantly, the swimming motion of these photolithography-fabricated was different in a reversed rotating magnetic field. Moreover, the effect of the thickness of the microswimmer was studied and it was found that the microswimmer with a thickness equal to half of the critical size had the fastest swimming velocity. Theoretical calculations based on the multipole expansion method have been performed to verify the experimental data and discussions have been made. Finally, a zigzag swimming strategy is pointed out with experiments for swimming on a substrate by taking the advantage of the unidirectional property of the achiral microswimmers.

## Data Availability

Not applicable.

## Supplementary Materials

The following supporting information can be downloaded at: https://www.mdpi.com/article/10.3390/mi13111965/s1.
